# Supplementary material for: Transdifferentiation of Human Fibroblasts into Skeletal Muscle Cells: Optimization and Assembly into Engineered Tissue Constructs through Biological Ligands
Source: Biology (Basel). 2021 Jun 16;10(6):539. doi: 10.3390/biology10060539 (PMC8235639; doi:10.3390/biology10060539)
Supplement: Supplementary file 1 [file biology-10-00539-s001.zip › biology-1222602-supplementary.pdf]

Table S1: Description of Biological Ligands administered in screening of differentiated skeletal muscle cells. Skeletal muscle cells were differentiated (C2C12s and Human Skeletal Myoblasts) or transdifferentiated human dermal fibroblasts and subsequently screened with 1 ng/ml and 10 ng/ml biological ligands. These proteins have been described as to play a vital role in myogenic signaling pathways that result in developmental and regenerative response.

Ligand Protein	Signaling Pathway	Myogenic Function
**Follistatin (Fs)**	TGF-β	Inhibition and sequestering of activin [1,2], stimulation of follicle stimulating hormone, promotion of proliferation and differentiation [2,3]; promotes skeletal muscle doubling, through hypertrophy and hyperplasia [4–6]
**Myostatin/Growth Differentiation Factor 8 (GDF8)**	TGF-β	A myokine, autocrine protein released by skeletal muscle cells [3,7,8], activin binding protein, regulator an skeletal muscle development, when silenced mice experienced muscular doubling [9–11]
**Fibroblast Growth Factor 2 (FGF2)**	PI3K/AKT/mTOR and MAPK/ERK	Promotes cell growth and development, tissue regeneration and increases proliferation, inhibitor of myogenic differentiation [12–15]
**GDF11**	TGF-β	Blood expression levels are elevated at birth and declines with age [16]; Conflicting results regarding myogenesis and muscle aging; overexpression in old mice increased cardiac and skeletal muscle regeneration [16–18], expression levels elevated in active individuals compared to non-active [17]; irreproducible and contradictory results with exposure to old mice cardiac cells, *in vitro* exposure to human donor skeletal muscle cells decreased differentiation [19–23]
**GDF15**	TGF-β	Upregulated during injury, regulates inflammatory response and macrophage invasion, promotes regeneration and anti-inflammatory effects [24–26]
**human Growth Hormone (hGH)**	JAK-STAT, MAPK/ERK, and PI3k/AKT/mTOR	Improves skeletal muscle proliferation and differentiation; increases skeletal muscle mass, however prolonged exposure induces tumorigenesis [27–31]
**Thymosin β (TMSB4X)**	NF-κB and PI3K/AKT/mTOR	Sequestering of G-actin [32], promotes skeletal muscle oriented F-actin [33], anti-inflammatory response, improve repair, regeneration and angiogenesis [34–37]
**Bone Morphogenetic Protein 4 (BMP4)**	TGF-β	Induction of proliferation, promoting differentiation towards osteogenic and chondrogenic cell lineages, inhibition of MyoD1[38–41]
**BMP7**	TGF-β	Inhibition of skeletal muscle differentiation, skeletal muscle apoptosis, induction of osteogenesis [3,6,42]
**Interleukin 6 (IL6)**	p38 MAPK and JAK-STAT	Anti-inflammatory myokine, promotes wound healing and myogenesis, induction of uptake of glucose, significant blood levels elevated during exercise, *in vitro* exposure induced myoblast differentiation, in addition to proliferation during injury[39,43–46]
**Tumor Necrosis Factor Alpha (TNF-α)**	NF-κB, MAPK/ERK, and PI3K/AKT/mTOR	Inflammatory cytokine, inducing muscle wasting and apoptosis, C2C12 exposure demonstrated arrested differentiation and progressive apoptosis [47–52]

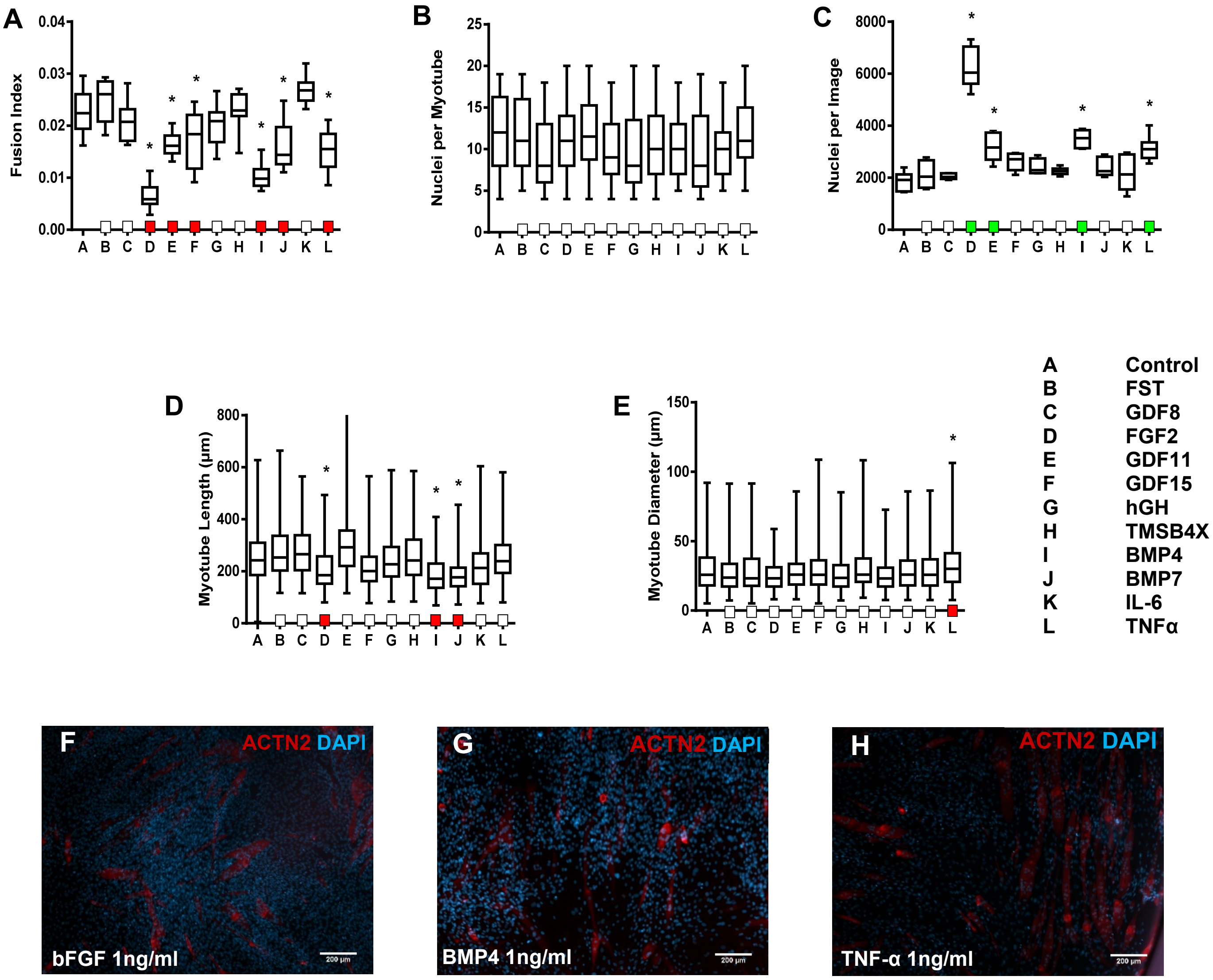

Figure S1: Ligand screening of differentiated mouse skeletal muscle cells C2C12 at 1 ng/ml. C2C12 skeletal muscle cells were subject to 1 ng/ml ligands, subsequent of 7 days of differentiation, stained with anti-ACTN2, anti-MF20 cytoskeletal stains and DAPI nuclear stain 7 days later. (A) Fusion index was delineated from myonucleation level divided by total number of nuclei (n=12, mean + SD, "*" denotes significance $p<0.05$ against control). (B) multinucleation of myotubes at 1 ng/ml was measured from C2C12 (n>31, mean + SD, "*" denotes significance $p<0.05$, against control). (C) Nuclear density of both skeletal muscle cells was quantified with number of nuclei stained ($DAPI^+$) (n=6, mean + SD, "*" denotes significance $p<0.05$, against control). (D) Myotube length (µm) was measured (n> 135, mean + SD, "*" denotes significance $p<0.05$, against control) along with (E) diameter (µm) of cells, per ligand (n> 160, mean + SD, "*" denotes significance $p<0.05$ against control). Cells exposed to TNF-α showed increased myotube development compared to control. (F) Expression of $ACTN2^+$ and $DAPI^+$ skeletal myotubes C2C12s. Nuclear expansion was improved with exposure to bFGF, additionally a reduction of differentiation and prolifereation of skeletal muscle cells under the influence of BMP4 was observed, and enhancement of myotube morphological features (length and diameter) with the

administration of TNF-α. C2C12s showed greater propensity towards the observed effects at higher concentrations of the aforementioned ligands (bFGF, BMP4, TNF-α).

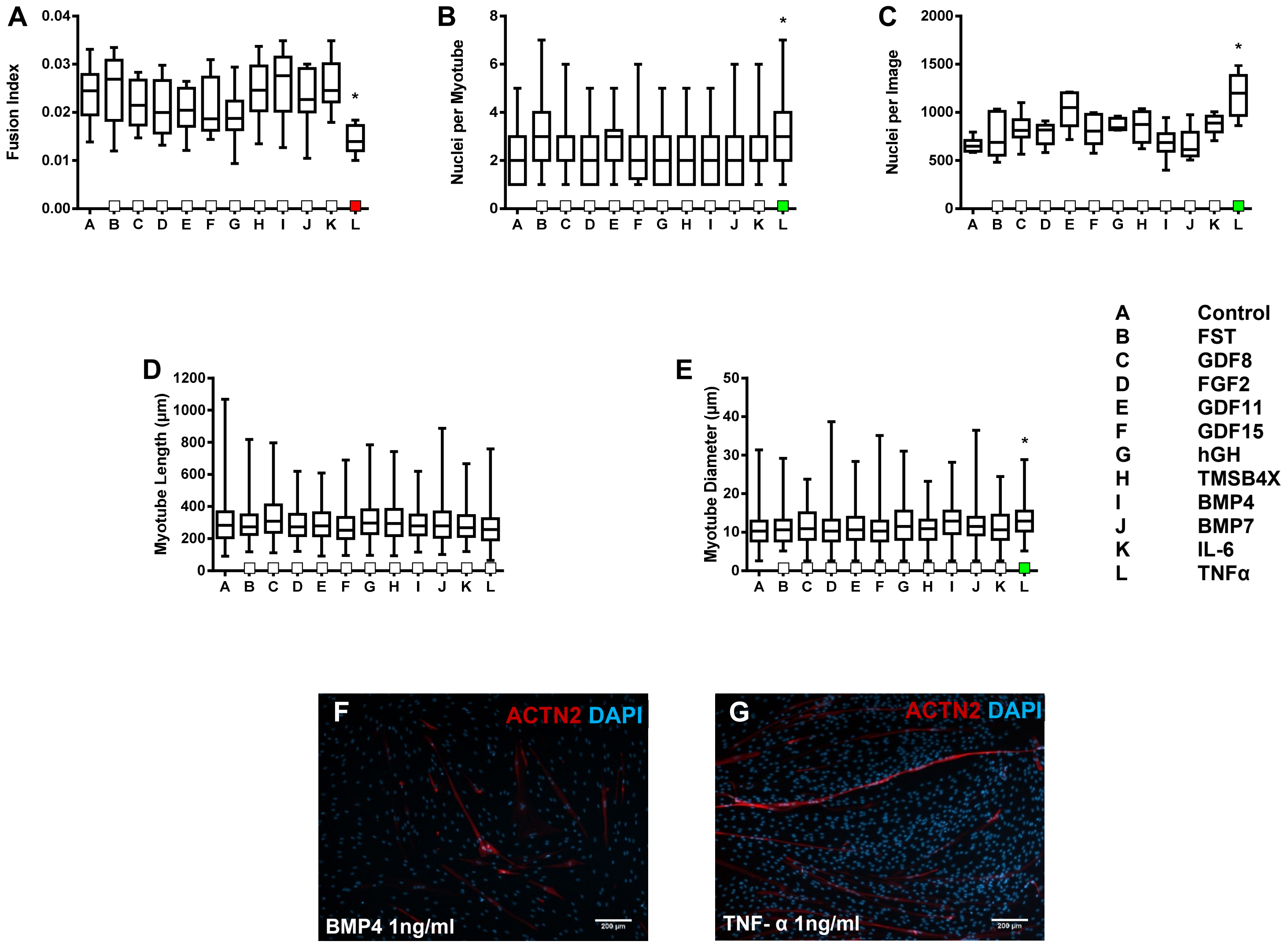

Figure S2: Human Skeletal Myoblasts HSkM screening of 1 ng/ml biological ligand. Initially cells were differentiated for a period of 7 days before the exposure to biological ligands, involved in skeletal muscle regulation and wound remodeling, for an additional 7-day period, and subsequently immunostained to identify effect of myotube differentiation/proliferation parameters. (A) Fusion index was calculated by measuring myotube nuclear density divided by total nuclei (n=12, mean + SD, "*" denotes significance $p<0.05$ against control). (B) Myonucleation was determined following the screening of biological ligands exposure (n>45, mean + SD, "*" denotes significance $p<0.05$, against control). (C) Cell nuclear density was measured by counting $DAPI^+$ cells of each screening condition (n=6, mean + SD, "*" denotes significance $p<0.05$, against control). (D) Differentiated HSkM were morphologically evaluated with the measurement of

myotube length (μm) from ACTN2⁺ cells (n>120, mean + SD, “*” denotes significance $p<0.05$, against control) and (E) Myotube diameter (μm) (n>150, mean + SD, “*” denotes significance $p<0.05$, against control). (F) ACTN2 and DAPI staining indicated significant morphological variation in HSkM cells when exposed to TNF-α, in addition, increased multinucleation and the overall nuclear count, with respect to untreated HSkM.

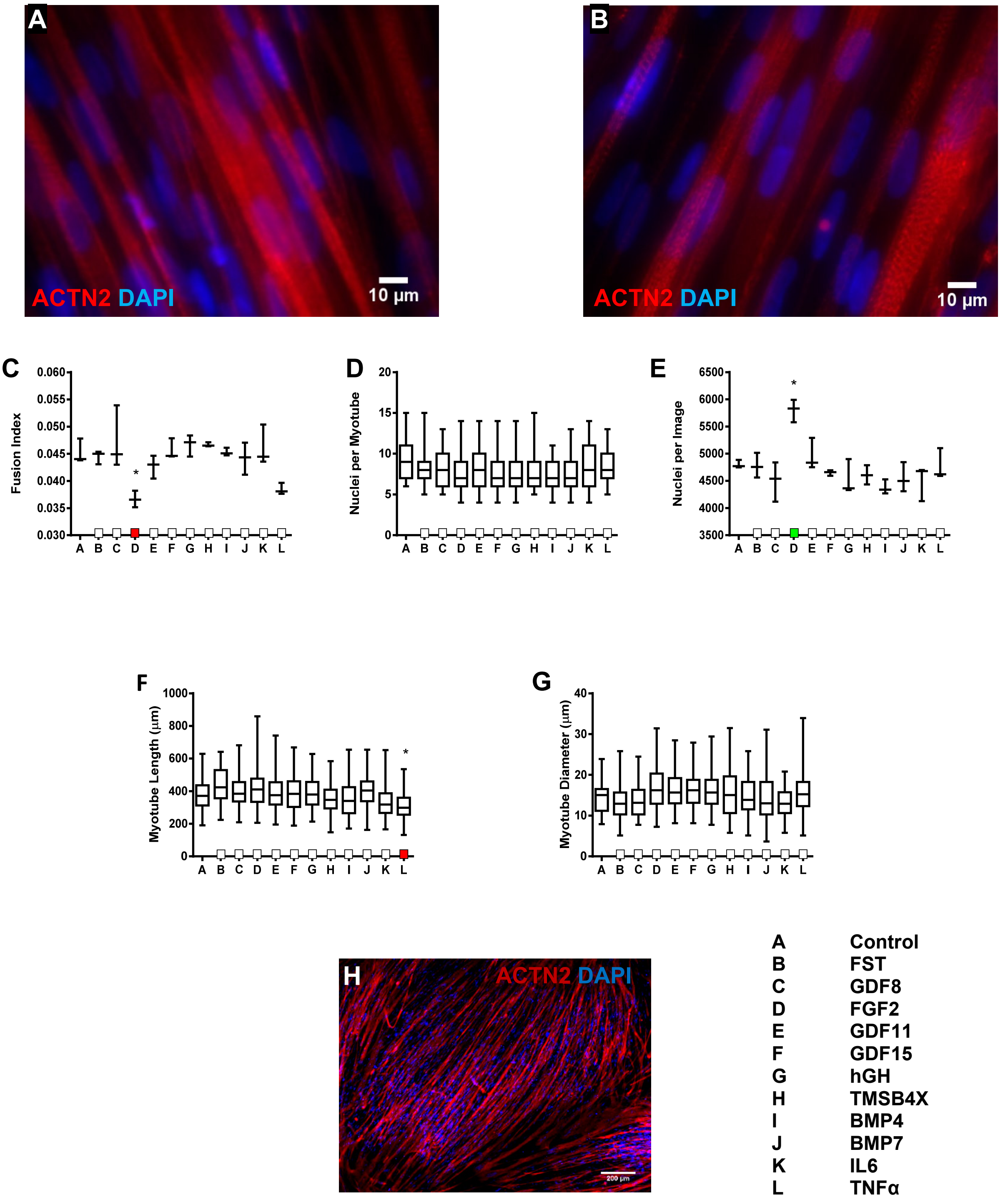

Figure S3: Biological ligand screening (1 ng/ml) of transdifferentiated human fibroblasts (tHFs). Transdifferentiation of HFs was induced via transduction of MYOD gene integration, prompting expression through doxycycline and SB43154 incorporation. Cells were induced to transdifferentiate for 7 days before administration of Dox and SB were suspended and the exposure of biological ligands commenced, for an additional 7 days. Immunohistochemistry of tHFs with ACTN2, MF20 and DAPI staining on the 14-day mark was utilized to evaluate the effect of biological ligands on skeletal muscle differentiation. (A) High resolution microscopy (60x) of untreated tHFs. (B) Cross-striation formed in cells treated with BMP4 (10 ng/ml) as observed from high magnification images. (C) tHf myotube fusion index was assessed by measuring myotube nuclei level vs total number of nuclei (n=3, mean + SD, "*" denotes significance $p<0.05$ against control). (D) Myotube nuclei count was quantified (n=30, mean + SD, "*" denotes significance $p<0.05$, against control) and (E) nuclear density $DAPI^+$ cells, including normal fibroblasts (n=3, mean + SD, "*" denotes significance $p<0.05$, against control). (F) Myotube length (μm) and (G) myotube diameter (μm) derived from $ACTN2^+$ cells (n>60, mean + SD, "*" denotes significance $p<0.05$, against control) (H) Nuclear expansion displayed significant increases without presenting improvement in differentiation parameters with bFGF treatment.

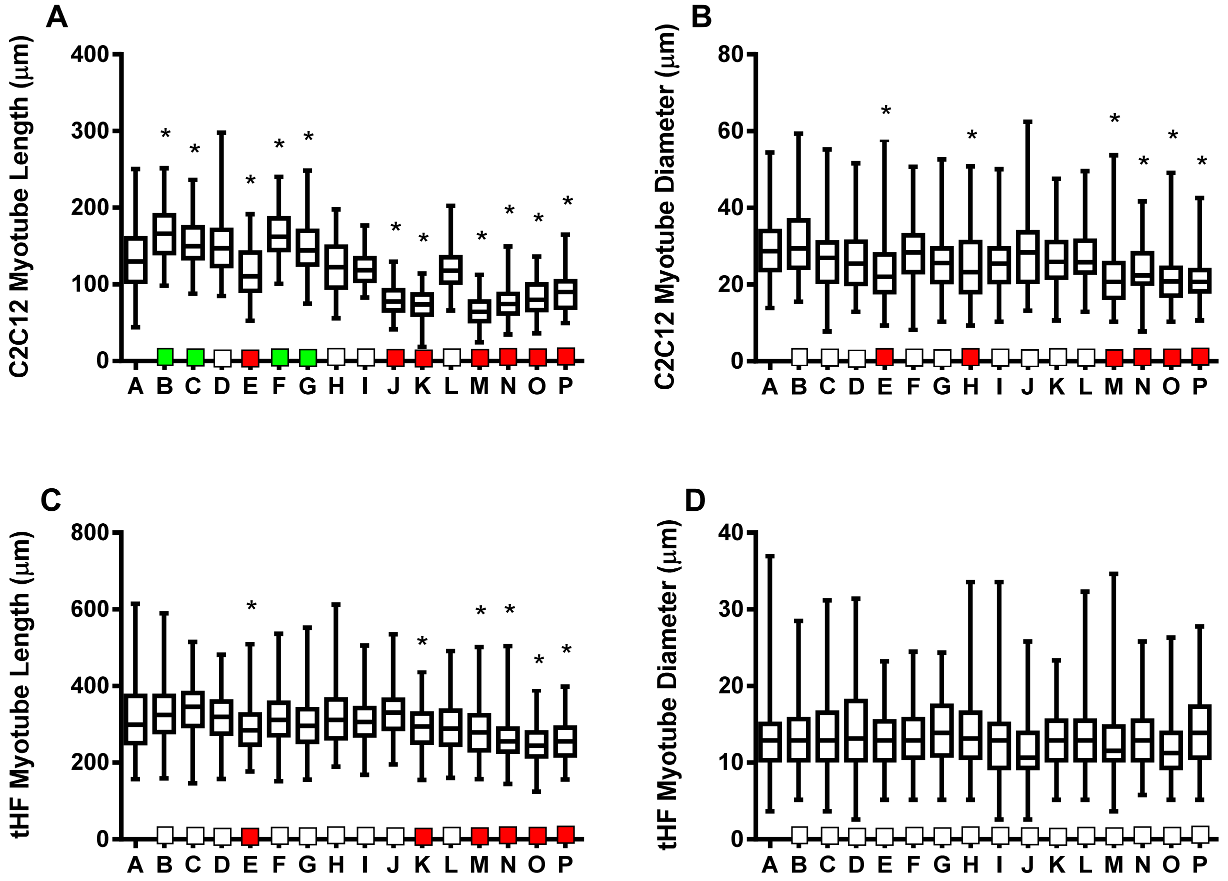

**A**	**Control**	**I**	**T+I**
**B**	**GDF11 (G)**	**J**	**T+F**
**C**	**TMSB4X (T)**	**K**	**I+F**
**D**	**IL6 (I)**	**L**	**G+T+I**
**E**	**TNFα (F)**	**M**	**G+T+F**
**F**	**G+T**	**N**	**G+I+F**
**G**	**G+I**	**O**	**T+I+F**
**H**	**G+F**	**P**	**G+T+I+F**

Figure S4: Myotube length and diameter of C2C12 and tHF cells exposed to combinations of GDF-11, TMSBX, IL-6, and TNF-α at 10 ng/ml. C2C12 and HF were respectively differentiated and transdifferentiated for seven days, and treated with combinations of GDF-11, TMSBX, IL-6, and TNF-α at 10 ng/ml for a total of 15 unique conditions, according to previously established protocol. Cells were quantified for fusion index, multinucleation, nuclear density per image, nuclear count of MYOD1 and Ki67 positive cells in figure 4 (C2C12s) and figure 5 (tHFs). (A) C2C12 myotube length (μm) was measured from cells expressing $ACTN^+$ ($n>80$, mean + SD, "*" denotes significance $p<0.05$, against control). (B) Quantification of C2C12 diameter (μm) of $ACTN^+$ C2C12s ($n>50$, mean + SD, "*" denotes significance $p<0.05$, against control). Cells exposed to TNF-α combinations showed significantly decreases compared to control cells (bar GF C2C12 length, TF and IF C2C12 diameter). (C) Myotube length (μm) of tHF was measured and tabulated ($n>130$, mean + SD, "*" denotes significance $p<0.05$, against control). (D)

Similarly, myotube diameter (μm) was determined from tHF cells that have also been measured for length (n>160, mean + SD, “*” denotes significance $p<0.05$, against control). TNF-α incorporation with other ligands resulted in large scale decrease in tHF length (bar GF and TF and IF combinations).

References

1. Lin, S.-Y.; Morrison, J.R.; Phillips, D.J.; de Kretser, D.M. Regulation of ovarian function by the TGF-beta superfamily and follistatin. *Reproduction* **2003**, *126*, 133–148, doi:10.1530/reprod/126.2.133.

2. Lee, S.-J.; Lee, Y.-S.; Zimmers, T. a; Soleimani, A.; Matzuk, M.M.; Tsuchida, K.; Cohn, R.D.; Barton, E.R. Regulation of muscle mass by follistatin and activins. *Mol. Endocrinol.* **2010**, *24*, 1998–2008, doi:10.1210/me.2010-0127.

3. Suryawan, A.; Frank, J.W.; Nguyen, H. V.; Davis, T.A. Expression of the TGF-?? family of ligands is developmentally regulated in skeletal muscle of neonatal rats. *Pediatr. Res.* **2006**, *59*, 175–179, doi:10.1203/01.pdr.0000196718.47935.6e.

4. Lee, S.J. Quadrupling muscle mass in mice by targeting TGF-?? signaling pathways. *PLoS One* **2007**, *2*, doi:10.1371/journal.pone.0000789.

5. Winbanks, C.E.; Weeks, K.L.; Thomson, R.E.; Sepulveda, P. V.; Beyer, C.; Qian, H.; Chen, J.L.; Allen, A.M.; Lancaster, G.I.; Febbraio, M.A.; et al. Follistatin-mediated skeletal muscle hypertrophy is regulated by Smad3 and mTOR independently of myostatin. *J. Cell Biol.* **2012**, *197*, 997–1008, doi:10.1083/jcb.201109091.

6. Amthor, H.; Christ, B.; Rashid-Doubell, F.; Kemp, C.F.; Lang, E.; Patel, K. Follistatin regulates bone morphogenetic protein-7 (BMP-7) activity to stimulate embryonic muscle growth. *Dev. Biol.* **2002**, *243*, 115–27, doi:10.1006/dbio.2001.0555.

7. Lee, S.-J.; McPherron, A.C. Myostatin and the control of skeletal muscle mass: Commentary. *Curr. Opin. Genet. Dev.* **1999**, *9*, 604–607, doi:10.1016/S0959-437X(99)00004-0.

8. Zhao, B.L.; Kollias, H.D.; Wagner, K.R. Myostatin directly regulates skeletal muscle fibrosis. *J. Biol. Chem.* **2008**, *283*, 19371–19378, doi:10.1074/jbc.M802585200.

9. McPherron, A.C.; Lawler, A.M.; Lee, S.-J. Regulation of skeletal muscle mass in mice by a new TGF-p superfamily member. *Nature* 1997, *387*, 83–90.

10. McPherron, A.C.; Lee, S.J. Double muscling in cattle due to mutations in the myostatin gene. *Proc. Natl. Acad. Sci. U. S. A.* **1997**, *94*, 12457–61, doi:10.1073/pnas.94.23.12457.

11. Kollias, H.D.; Mcdermott, J.C. Transforming growth factor-B and myostatin signaling in skeletal muscle. *J. Appl. Physiol.* **2008**, *104*, 579–587, doi:10.1152/japplphysiol.01091.2007.

12. Johnson, S.E.; Allen, R.E. Activation of skeletal muscle satellite cells and the role of fibroblast growth factor receptors. *Exp. Cell Res.* 1995, *219*, 449–453.

13. Clarke, M.S.; Feeback, D.L. Mechanical load induces sarcoplasmic wounding and FGF release in differentiated human skeletal muscle cultures. *Faseb J* **1996**, *10*, 502–509.

14. Johnson, S.E.; Allen, R.E. The effects of bFGF, IGF-I, and TGF-beta on RMo skeletal muscle cell proliferation and differentiation. *Exp. Cell Res.* **1990**, *187*, 250–254.

15. Allen, R.E.; Boxhorn, L.K. Regulation of skeletal muscle satellite cell proliferation and differentiation by transforming growth factor-beta, insulin-like growth factor I, and fibroblast growth factor. *J. Cell. Physiol.* **1989**, *138*, 311–315, doi:10.1002/jcp.1041380213.

16. Loffredo, F.S.; Steinhauser, M.L.; Jay, S.M.; Gannon, J.; Pancoast, J.R.; Yalamanchi, P.; Sinha, M.; Dall'Osso, C.; Khong, D.; Shadrach, J.L.; et al. Growth differentiation factor 11 is a circulating factor that reverses age-related cardiac hypertrophy. *Cell* **2013**, *153*, 828–839, doi:10.1016/j.cell.2013.04.015.

17. Sinha, M.; Jang, Y.C.; Oh, J.; Khong, D.; Wu, E.Y.; Manohar, R.; Miller, C.; Regalado, S.G.; Loffredo, F.S.; Pancoast, J.R.; et al. Restoring systemic GDF11 levels reverses age-related dysfunction in mouse skeletal muscle. *Science* **2014**, *344*, 649–52, doi:10.1126/science.1251152.

18. Du, G.-Q.; Shao, Z.-B.; Wu, J.; Yin, W.-J.; Li, S.-H.; Wu, J.; Weisel, R.D.; Tian, J.-W.; Li, R.-K. Targeted myocardial delivery of GDF11 gene rejuvenates the aged mouse heart and enhances myocardial regeneration after ischemia–reperfusion injury. *Basic Res. Cardiol.* **2017**, *112*, 7, doi:10.1007/s00395-016-0593-y.

19. Hammers, D.W.; Merscham-Banda, M.; Hsiao, J.Y.; Engst, S.; Hartman, J.J.; Sweeney, H.L. Supraphysiological levels of GDF11 induce striated muscle atrophy. *EMBO Mol. Med.* **2017**, e201607231, doi:10.15252/emmm.201607231.

20. Egerman, M.A.; Cadena, S.M.; Gilbert, J.A.; Meyer, A.; Nelson, H.N.; Swalley, S.E.; Mallozzi, C.; Jacobi, C.; Jennings, L.L.; Clay, I.; et al. GDF11 Increases with Age and Inhibits Skeletal Muscle Regeneration. *Cell Metab.* **2015**, *22*, 164–174, doi:10.1016/j.cmet.2015.05.010.

21. Smith, S.C.; Zhang, X.; Zhang, X.; Gross, P.; Starosta, T.; Mohsin, S.; Franti, M.; Gupta, P.; Hayes, D.; Myzithras, M.; et al. GDF11 Does Not Rescue Aging-Related Pathological Hypertrophy. *Circ. Res.* **2015**, *117*, 926–932, doi:10.1161/CIRCRESAHA.115.307527.

22. Walker, R.G.; Poggioli, T.; Katsimpardi, L.; Buchanan, S.M.; Oh, J.; Wattrus, S.; Heidecker, B.; Fong, Y.W.; Rubin, L.L.; Ganz, P.; et al. Biochemistry and Biology of GDF11 and Myostatin. *Circ. Res.* **2016**, *118*, 1125–1142, doi:10.1161/CIRCRESAHA.116.308391.

23. Schafer, M.J.; Atkinson, E.J.; Vanderboom, P.M.; Kotajarvi, B.; White, T.A.; Moore, M.M.; Bruce, C.J.; Greason, K.L.; Suri, R.M.; Khosla, S.;

et al. Quantification of GDF11 and Myostatin in Human Aging and Cardiovascular Disease. *Cell Metab.* **2016**, *23*, 1207–1215, doi:10.1016/j.cmet.2016.05.023.

24. Kempf, T.; Zarbock, A.; Widera, C.; Butz, S.; Stadtmann, A.; Rossaint, J.; Bolomini-Vittori, M.; Korf-Klingebiel, M.; Napp, L.C.; Hansen, B.; et al. GDF-15 is an inhibitor of leukocyte integrin activation required for survival after myocardial infarction in mice. *Nat. Med.* **2011**, *17*, 581–588, doi:10.1038/nm.2354.

25. Kalli, M.; Minia, A.; Pliaka, V.; Fotis, C.; Alexopoulos, L.G.; Stylianopoulos, T. Solid stress-induced migration is mediated by GDF15 through Akt pathway activation in pancreatic cancer cells. *Sci. Rep.* **2019**, *9*, 1–12, doi:10.1038/s41598-018-37425-6.

26. Unsicker, K.; Spittau, B.; Krieglstein, K. The multiple facets of the TGF-β family cytokine growth/differentiation factor-15/macrophage inhibitory cytokine-1. *Cytokine Growth Factor Rev.* **2013**, *24*, 373–384, doi:10.1016/j.cytogfr.2013.05.003.

27. Clark, W.; Kendall, M.J. Growth hormone treatment for growth hormone deficient adults. *J. Clin. Pharm. Ther.* **1996**, *21*, 367–372.

28. Cuneo, R.; Salomon, F.; Mark Wiles, C.; Sonksen, P.H. Skeletal Muscle Performance in Adults with Growth Hormone Deficiency. *Horm. Res. Paediatr.* **1990**, *Vol 33*.

29. Isgaard, J.; Nilsson, a; Vikman, K.; Isaksson, O.G. Growth hormone regulates the level of insulin-like growth factor-I mRNA in rat skeletal muscle. *J. Endocrinol.* **1989**, *120*, 107–12.

30. Yarasheski, K.E.; Campbell, J.A.; Smith, K.; Rennie, M.J.; Holloszy, J.O.; Bier, D.M. Effect of growth hormone and resistance exercise on muscle growth in young men. *Am J Physiol* **1992**, *262*, E261-7.

31. Molitch, M.E. Growth hormone treatment in adults with growth hormone deficiency: the transition. *J Endocrinol Invest* **2011**, *34*, 150–154, doi:7483 [pii]\r10.3275/7483.

32. Cassimeris, L.; Safer, D.; Nachmias, V.T.; Zigmond, S.H. Thymosin beta4 sequester the majority of G-actin in resting human polymorphonuclear leukocytes. *J. Cell. Biol.* **1992**, *119*, 1261-1270.

33. Vancompernolle, K.; Goethals, M.; Huet, C.; Louvard, D.; Vandekerckhove, J. G- substitution the thymosin. **1992**, *1*, 4739–4746.

34. Smart, N.; Bollini, S.; Dubé, K.N.; Vieira, J.M.; Zhou, B.; Davidson, S.; Yellon, D.; Riegler, J.; Price, A.N.; Lythgoe, M.F.; et al. De novo cardiomyocytes from within the activated adult heart after injury. *Nature* **2011**, *474*, 640–4, doi:10.1038/nature10188.

35. Philp, D.; Goldstein, A.L.; Kleinman, H.K. Thymosin β4 promotes angiogenesis, wound healing, and hair follicle development. *Mech. Ageing Dev.* **2004**, *125*, 113–115, doi:10.1016/j.mad.2003.11.005.

36. Smart, N.; Risebro, C.A.; Melville, A.A.D.; Moses, K.; Schwartz, R.J.; Chien, K.R.; Riley, P.R. Thymosin beta4 induces adult epicardial progenitor mobilization and neovascularization. *Nature* **2007**, *445*, 177–182, doi:10.1038/nature05383.

37. Spurney, C.F.; Cha, H.J.; Sali, A.; Pandey, G.S.; Pistilli, E.; Guerron, A.D.; Gordish-Dressman, H.; Hoffman, E.P.; Nagaraju, K. Evaluation of skeletal and cardiac muscle function after chronic administration of thymosin ??-4 in the dystrophin deficient mouse. *PLoS One* **2010**, *5*, 16–18, doi:10.1371/journal.pone.0008976.

38. Ono, Y.; Calhabeu, F.; Morgan, J.E.; Katagiri, T.; Amthor, H.; Zammit, P.S. BMP signalling permits population expansion by preventing premature myogenic differentiation in muscle satellite cells. *Cell Death Differ.* **2011**, *18*, 222–34, doi:10.1038/cdd.2010.95.

39. Pedersen, B.K.; Akerstrom, T.C.; Nielsen, A.R.; Fischer, C.P.; Amthor, H.; Christ, B.; Patel, K. Role of myokines in exercise and metabolism. *J Appl Physiol* **2007**, *126*, 1041–53, doi:10.1152/japplphysiol.00080.2007.

40. Jackson, W.M.; Aragon, A.B.; Onodera, J.; Koehler, S.M.; Ji, Y.; Bulken-Hoover, J.D.; Vogler, J.A.; Tuan, R.S.; Nesti, L.J. Cytokine expression in muscle following traumatic injury. *J. Orthop. Res.* **2011**, *29*, 1613–1620, doi:10.1002/jor.21354.

41. Clever, J.L.; Sakai, Y.; Wang, R. a; Schneider, D.B. Inefficient skeletal muscle repair in inhibitor of differentiation knockout mice suggests a crucial role for BMP signaling during adult muscle regeneration. *Am. J. Physiol. Cell Physiol.* **2010**, *298*, C1087–C1099, doi:10.1152/ajpcell.00388.2009.

42. Komaki, M.; Asakura, a; Rudnicki, M. a; Sodek, J.; Cheifetz, S. MyoD enhances BMP7-induced osteogenic differentiation of myogenic cell cultures. *J. Cell Sci.* **2004**, *117*, 1457–1468, doi:10.1242/jcs.00965.

43. Vargha, A.; Delaney, H.D.; Silberstein, L.; Webster, S.G.; Travis, M.; Blau, H.M.; Koh, G.Y.; Klug, M.G.; Soonpaa, M.H.; Field, L.J.; et al. Cell surface fibroblast growth factor and epidermal growth factor receptors are permanently lost during skeletal muscle terminal differentiation in culture. *Cell* **2008**, *23*, 463–408, doi:10.3102/10769986023002170.

44. Pedersen, B.K.; Akerstrom, T.C.; Nielsen, A.R.; Fischer, C.P. Role of myokines in exercise and metabolism. *J Appl Physiol* **2007**, *103*, 1093–1098, doi:10.1152/japplphysiol.00080.2007.

45. Serrano, A.L.; Baeza-Raja, B.; Perdiguero, E.; Jardí, M.; Muñoz-Cánoves, P. Interleukin-6 Is an Essential Regulator of Satellite Cell-Mediated Skeletal Muscle Hypertrophy. *Cell Metab.* **2008**, *7*, 33–44, doi:10.1016/j.cmet.2007.11.011.

46. Febbraio, M.A.; Hiscock, N.; Sacchetti, M.; Fischer, C.P.; Pedersen, B.K. Interleukin-6 Is a Novel Factor Mediating Glucose Homeostasis During Skeletal Muscle Contraction. *Diabetes 53*, 1643–1648.

47. Timper, K.; Denson, J.L.; Steculorum, S.M.; Heilinger, C.; Engström-Ruud, L.; Wunderlich, C.M.; Rose-John, S.; Wunderlich, F.T.; Brüning, J.C.; Li, G.; et al. Exercise and IL-6 infusion inhibit endotoxin-induced TNF-alpha production in humans. *Am. J. Physiol. Integr. Comp. Physiol.* **2017**, *10*, 884–886, doi:10.1152/ajpregu.00373.2016.

48. Sen, C.K.; Khanna, S.; Reznick, A.Z.; Roy, S.; Packer, L. Glutathione regulation of tumor necrosis factor-α-induced NF-κB activation in skeletal muscle-derived L6 cells. *Biochem. Biophys. Res. Commun.* **1997**, *237*, 645–649, doi:10.1006/bbrc.1997.7206.

49. Li, Y.P.; Schwartz, R.J.; Waddell, I.D.; Holloway, B.R.; Reid, M.B. Skeletal muscle myocytes undergo protein loss and reactive oxygen-mediated NF-kappaB activation in response to tumor necrosis factor alpha. *FASEB J.* **1998**, *12*, 871–80.

50. Jackman, R.W.; Kandarian, S.C. The molecular basis of skeletal muscle atrophy. *Am. J. Physiol. Cell Physiol.* **2004**, *287*, C834-43, doi:10.1152/ajpcell.00579.2003.

51. Hunter, R.B.; Stevenson, E.; Koncarevic, A.; Mitchell-Felton, H.; Essig, D. a; Kandarian, S.C. Activation of an alternative NF-kappaB pathway in skeletal muscle during disuse atrophy. *FASEB J.* **2002**, *16*, 529–538, doi:10.1096/fj.01-0866com.

52. Reid, M.B.; Li, Y.P. Tumor necrosis factor-?? and muscle wasting: A cellular perspective. *Respir. Res.* **2001**, *2*, 269–272, doi:10.1186/rr67.
